# Cognitive brain responses during circadian wake-promotion: evidence for sleep-pressure-dependent hypothalamic activations

**DOI:** 10.1038/s41598-017-05695-1

**Published:** 2017-07-17

**Authors:** Carolin F. Reichert, Micheline Maire, Virginie Gabel, Antoine U. Viola, Thomas Götz, Klaus Scheffler, Markus Klarhöfer, Christian Berthomier, Werner Strobel, Christophe Phillips, Eric Salmon, Christian Cajochen, Christina Schmidt

**Affiliations:** 1 Centre for Chronobiology, Psychiatric Hospital of the University of Basel, Basel, Switzerland; 2 Transfaculty Research Platform Molecular and Cognitive Neurosciences, University of Basel, Basel, Switzerland; 3 PPRS, Paris, France; 4 Department of Psychiatry, Public Health Office, Frankfurt am Main, Germany; 5 Max-Planck Institute for Biological Cybernetics, Tübingen, Germany; 6 Department of Medical Radiology, MR-Physics, University of Basel, Basel, Switzerland; 7 PHYSIP, Paris, France; 8 Respiratory Medicine, Department of Internal Medicine, University Hospital Basel, Basel, Switzerland; 9 GIGA-CRC, In Vivo Imaging Unit, University of Liège, Liège, Belgium

## Abstract

The two-process model of sleep-wake regulation posits that sleep-wake-dependent homeostatic processes interact with the circadian timing system to affect human behavior. The circadian timing system is fundamental to maintaining stable cognitive performance, as it counteracts growing homeostatic sleep pressure during daytime. Using magnetic resonance imaging, we explored brain responses underlying working memory performance during the time of maximal circadian wake-promotion under varying sleep pressure conditions. Circadian wake-promoting strength was derived from the ability to sleep during an evening nap. Hypothalamic BOLD activity was positively linked to circadian wake-promoting strength under normal, but not under disproportionally high or low sleep pressure levels. Furthermore, higher hypothalamic activity under normal sleep pressure levels predicted better performance under sleep loss. Our results reappraise the two-process model by revealing a homeostatic-dose-dependent association between circadian wake-promotion and cognition-related hypothalamic activity.

## Introduction

In humans, circadian and sleep-wake homeostatic processes interact to determine neurobehavioral performance over the 24-hour day^1^. Homeostatic sleep pressure accumulates with increasing time spent awake and dissipates with the opportunity to sleep^2^. The suprachiasmatic nuclei of the anterior hypothalamus are considered to be the brain’s circadian master clock, which is critically involved in the organization of sleep and wakefulness at different times of day. The circadian wake-drive is at its maximum 2–3 hours before habitual bedtime^3, 4^. This time window of maximal circadian wake-promotion has been characterized as the wake-maintenance zone (WMZ). The function of the WMZ is to efficiently counteract detrimental effects of homeostatic sleep pressure accumulating during wakefulness. Lacking or mistimed circadian wake-promotion affects state stability during wakefulness^5, 6^ and is associated with deterioration in neurobehavioral performance, particularly when sleep propensity is high^7, 8^. Accumulating evidence from both animal and human experiments indicates that the magnitude of circadian output signalling depends on homeostatic sleep pressure levels^7–14^ and vice versa^15–17^. It has been suggested that increasing homeostatic sleep pressure attenuates the strength of circadian wake promotion^18^.

**Figure 1:**
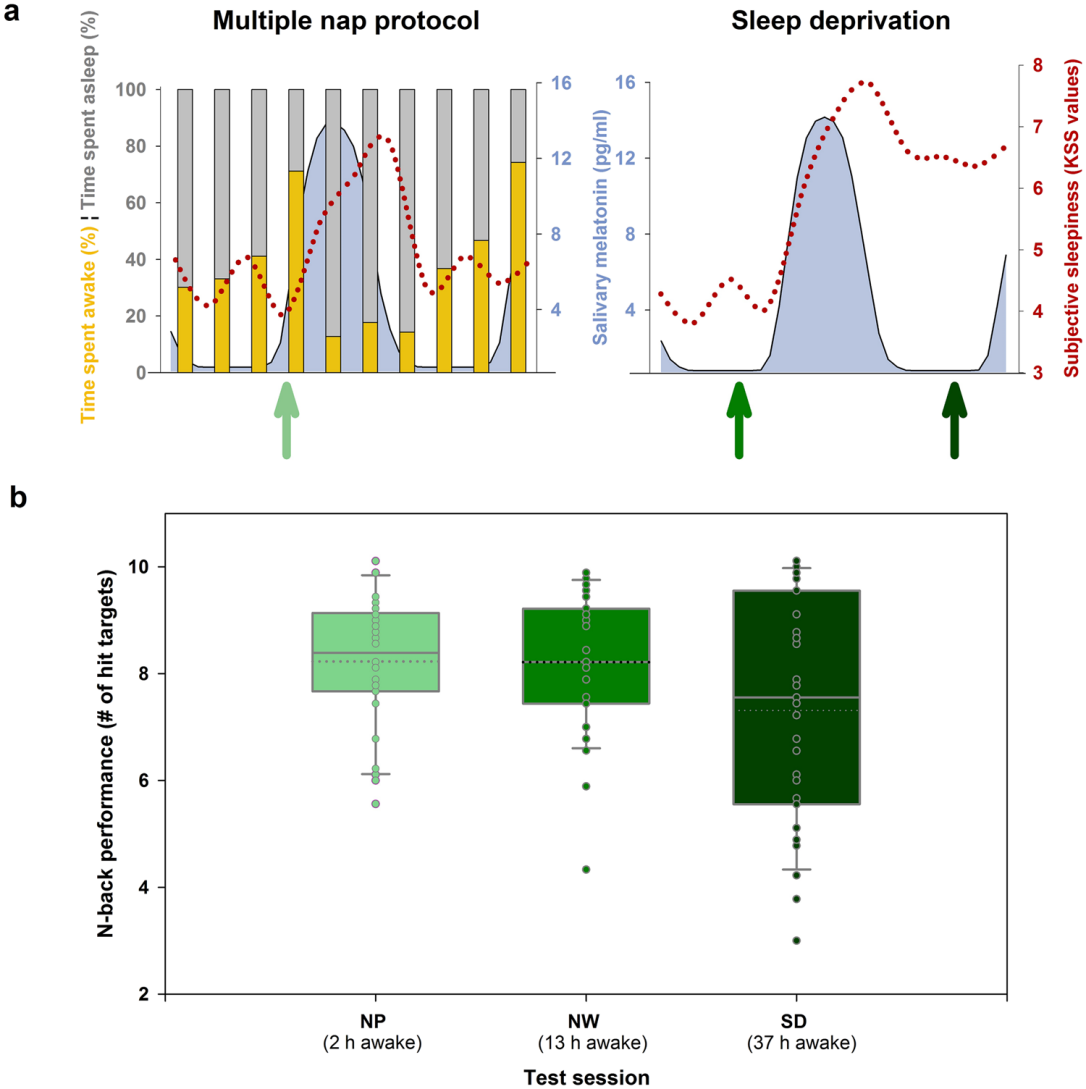
Effects of multiple napping and sleep deprivation. (**a**) In the 40-hour multiple nap protocol (NP), sleep efficiency was assessed over the 24-hour cycle. The ability to initiate and maintain sleep (nap sleep efficiency, grey bars, depicted as the percentage of sleep duration [all sleep stages combined] per total nap time) continuously decreased across daytime with lowest values (29%) in the late evening, while highest nap sleep efficiency (82–87%) was attained during the biological night (the latter indicated by melatonin secretion). In NP, subjective sleepiness (red dotted line) was low during daytime and high during night-time and early morning hours. A similar time course was observed during the 40-hour total sleep deprivation (SD). However, as sleep pressure levels accumulated in SD, an increase in sleepiness particularly after 24 hours of time into the protocol was observed when compared to NP. fMRI scanning sessions were scheduled in the late evening (green arrows), when wakefulness was strongly promoted by the circadian system. Participants were scanned once in the NP and two times in the sleep deprivation protocol (after 13 hours of wakefulness [i.e., under normal sleep pressure, referred to as normal waking (NW)] and 37 hours of wakefulness [i.e., under high sleep pressure, referred to as SD]). (**b**) Box plots illustrate median (solid line) and mean (dotted line) performance in the n-back across the scanning sessions, i.e., after different times awake before data acquisition. Mean performance was particularly impaired after 37 hours of continuous wakefulness (SD), mirroring the negative impact of high sleep pressure on working memory performance. Individual data are shown as dots.

The two-process model of circadian and homeostatic sleep-wake regulation has been linked to models of hypothalamic and brainstem neural activity, introducing a physiological basis for the wake-sleep switch^19, 20^. It is well established that hypothalamic nuclei are major determinants of sleep and wake timing^21, 22^. However, it remains unclear whether and under which specific conditions hypothalamic outputs are involved in the timing and control of human neurobehavioral functions. We previously reported a negative link between homeostatic sleep pressure build-up and task-related anterior hypothalamic blood-oxygen-level-dependent (BOLD) signals, when probing sustained attention in the evening hours in extreme chronotypes^23^. This result indicates that a trait-like difference of homeostatic sleep pressure has an impact on task-related hypothalamic activity. Here we investigated whether changes in the level of sleep pressure affects the association between circadian wake promotion and the hypothalamic BOLD signal. To do so, we explored cerebral correlates of cognitive performance during maximal circadian wake promotion under conditions of shortened and extended wakefulness. Under stringently controlled laboratory conditions, 31 volunteers (Table S1) underwent a 40-hour total sleep deprivation (SD) and multiple nap (NP) protocol in a within-subjects design. BOLD activity was recorded during the WMZ while performing a well-established working memory paradigm (n-back task)^24^. As depicted in Figure 1, the experimental setting thus allowed us to probe BOLD activity during the WMZ under 3 different homeostatic sleep pressure conditions: under low sleep pressure levels (after 2 hours of continuous wakefulness; NP condition), at the end of a normal waking day (NW, after 13 hours of continuous wakefulness, first test session of the SD protocol) as well as under high sleep pressure conditions (SD, after 37 hours of continuous wakefulness, last test session of the SD protocol). Similar to earlier studies^4, 25, 26^, the individual’s circadian wake-promoting strength was quantified by the ability to sleep during the WMZ with electro-encephalography (EEG nap sleep efficiency). We predicted that hypothalamic BOLD activity during successful task performance is linked to circadian wake-promoting strength, and that this link is modulated by sleep pressure levels. We expected this association to be attenuated under high sleep pressure conditions. On the other hand, assuming a monotonic dose-response relationship, the association should be amplified by disproportionally low sleep pressure levels induced by the NP condition.

## Results and Discussion

### Setting the context: characterization of the circadian wake-maintenance zone

The increase of sleep pressure during SD compared to NP manifested in a significant increase in EEG delta activity (EEG power in 0.7–4 Hz frequency range)^27^ during non-rapid eye movement (NREM) sleep (stages 2, 3, and 4) following SD (interaction night [baseline vs. recovery] × condition [SD vs. NP]): F_(1,23)_ = 12.10; *p* < 0.05; Cz-Pz derivation; mean ± standard deviation; baseline NP: 59.4 ± 27 µV^2^; baseline SD:64.1 ± 21 µV^2^; recovery NP 51.3 ± 22 µV^2^; recovery SD 83.3 ± 32 µV^2^; F_(1,23)_ = 21.9; p < 0.0001 for Fz derivation; mean ± standard deviation; baseline NP: 208.7 ± 124 µV^2^; baseline SD:199.1 ± 96 µV^2^; recovery NP 179.6 ± 72 µV^2^; recovery SD 314.5 ± 176 µV^2^; see supplemental Table S4 for full details). The magnetic resonance imaging (MRI) sessions were scheduled 13 h after habitual wake-up time, on average 2.2 hours before the evening onset of melatonin secretion (see methods), which hallmarks the end of the WMZ and opens the gate for sleep^25^. Figure 1 reveals that BOLD activity was measured before the night-time induced increase of subjective sleepiness for all sleep pressure conditions (Karolinska Sleepiness Scale, main effect of time F_(10,630)_ = 57.8; *p* < 0.001) and immediately preceded the start time of naps with the lowest sleep efficiency (local minimum, sleep efficiencies [percentage of sleep duration (all sleep stages combined) per total nap time] were significantly lower than in all other naps, Tuckey-Kramer adjusted post-hoc tests of main effect of time: *p*_all_ < 0.001).

### Working memory is jeopardized by sleep loss, also during the wake maintenance zone

We measured working memory (WM) and underlying cerebral correlates with a visual n-back task, frequently used in neuroimaging studies^24, 28^. Performance levels in the 3-back task during the WMZ under the three different sleep pressure conditions are illustrated in Figure 1b. As expected, performance (expressed as number of hits, i.e., true positive answers) was significantly worse under SD compared to NP (*p* = 0.006) and NW (*p* = 0.001), while performance between the NP and NW condition did not significantly differ (*p* = 0.776). At the cerebral level, hit targets in the 3-back task elicited the typical fronto-parietal pattern of cerebral BOLD activity^24^ (see Table S2). Activity in this representative pattern decreased in response to sleep loss compared to the nap condition (NP > SD) in the right dorso-lateral prefrontal cortex (x = 30, y = 46, z = 22; *p*_FWE_ = 0.02), the postcentral gyrus (x = −24, y = −58, z = 36; *p*_FWE_ = 0.015), and in a cluster in the cerebellum (x = −38, y = −48, z = −28; *p*_FWE_ = 0.029) extending to the fusiform gyrus and the inferior temporal lobe. Of these activations, dorso-lateral prefrontal activity tended to decrease as well under SD when compared to the normal waking day condition (NW > SD; x = 34, y = 52, z = 26, Z = 3.68, *p*_FWE_ = 0.072). Differences in the BOLD signal were positively correlated with differences in performance both between NP and SD and NW and SD (NP-SD: cerebellum *r* = 0.44, *p* = 0.013; NW-SD dorso-lateral prefrontal cortex *r* = 0.35, *p* = 0.036; all tests of significance: one-sided). Taken together, these results are in line with earlier findings on decreased frontal brain activity during working memory performance under sleep loss^29–31^. No significant increases in activity were observed in response to sleep loss (SD > NP *p*_FWE_ > 0.1 and SD > NW *p*_FWE_ > 0.1). Furthermore, low sleep pressure (NP) conditions did not induce any significant activity modulation compared to NW (NW > NP, NW < NP *p*_FWE_ > 0.1), adding to the literature that disproportionally low sleep pressure conditions do not seem to affect robust BOLD responses for successful task performance.

### Circadian wake-promoting strength and task-related hypothalamic BOLD activity are fine-tuned by homeostatic sleep pressure levels

Covariance analyses revealed that task-related BOLD signals were associated with sleep efficiency measured during a nap scheduled at a time of high circadian wake-promotion. High circadian wake-promotion was mirrored in the total absence of any sleep (i.e., sleep efficiency = 0) in 20% of the participants. Furthermore, the closer the nap was to the evening onset of melatonin secretion, the lower was sleep efficiency (*r* = −0.71; two-sided *p* < 0.001). This suggests that sleep propensity at this particular time window is phase locked to the evening onset of melatonin secretion.

**Figure 2:**
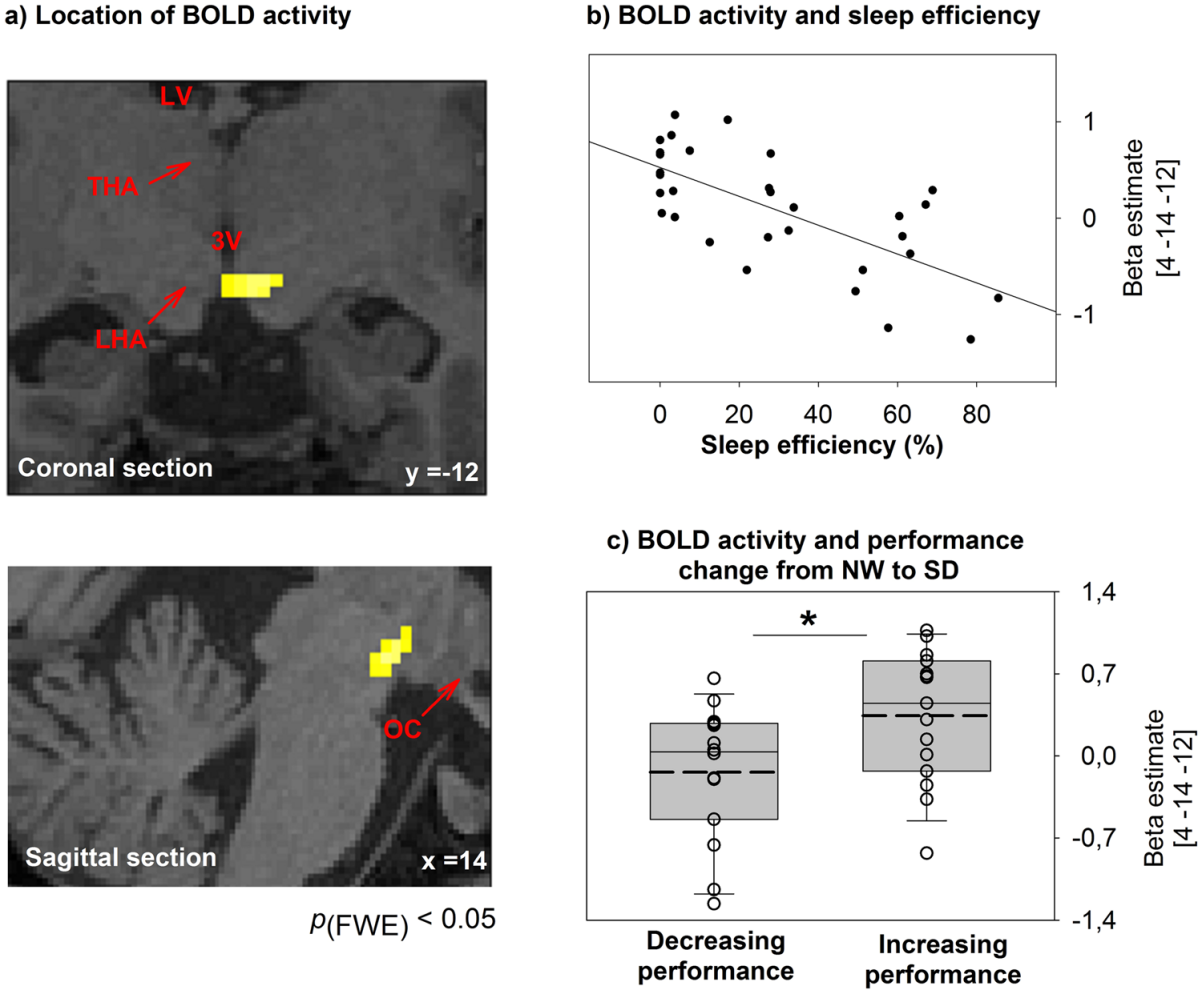
**(a)**﻿ The blood-oxygen-level-dependent (BOLD) hypothalamic signal, covarying with sleep efficiency in the late evening, is plotted on a structural image of a representative participant. BOLD activity was assessed during the late evening after 13 hours of continuous wakefulness. LV: lateral ventricle; THA: thalamus; 3V: third ventricle; LHT: lateral hypothalamus; OC: optic chiasm. **(b)** Correlation of hypothalamic BOLD activity after 13 hours of continuous wakefulness and sleep efficiency assessed during the multiple nap protocol in the evening (nap start 14 hours after regular wake-up time). Sleep efficiency (i.e., sleep duration per nap episode) refers to the ability to initiate and maintain sleep and is considered to reflect wake-promotion strength (i.e., higher sleep efficiency mirrors weaker wake-promotion). **(c)** Box plots illustrate the median (solid line) and mean (dashed line) hypothalamic BOLD signal according to performance changes from the first evening (13 hours of wakefulness, normal waking day, NW) to the second evening (SD, 37 hours of wakefulness) during sleep deprivation. Activity was lower in participants who decreased in performance from the first to the second evening compared to participants who had stable or increasing performance levels. The individual BOLD signal is shown as dots. *p < 0.05. *p*_FWE_: *p* after family-wise error correction.

**Figure 3:**
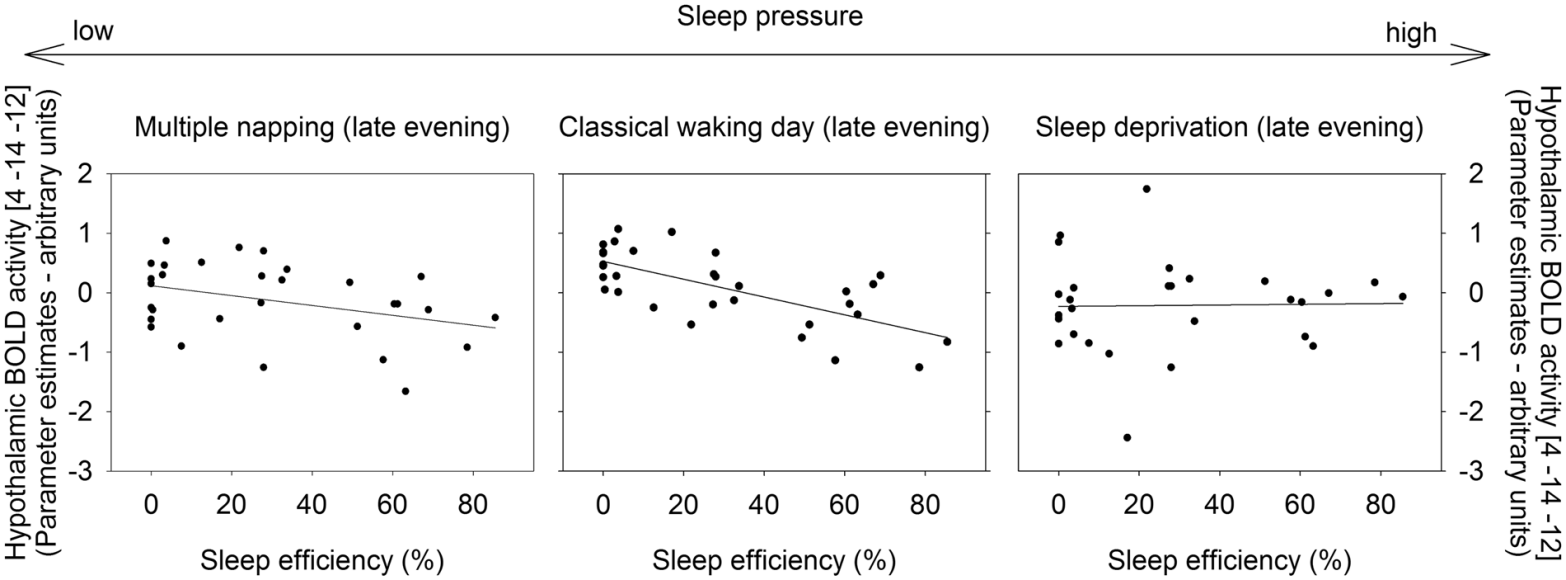
While hypothalamic blood-oxygen-level-dependent (BOLD) activity (depicted on the y-axis) was negatively associated with sleep efficiency (assessed in the late evening) after a classical waking day (middle panel), this relation was not significant during multiple napping (left panel; *p* = 0.05, not surviving correction for multiple comparisons) or sleep deprivation (right panel; *p* > 0.05). Multiple napping refers to conditions of low sleep pressure, as BOLD activity has been assessed after 2 h of continuous wakefulness. In comparison, the scan session after a night of sleep deprivation was scheduled at 37 hours of continuous wakefulness and thus took place﻿ under high sleep pressure levels.

Postero-lateral hypothalamic BOLD activity ([4 −12 −10], *p*_FWE_ = 0.007) was associated with sleep efficiency depending on sleep pressure (F_(1,84, 46,15)_ = 3.57, *p* = 0.039; Fig. 2a and b; see also Table S3). Under normal waking conditions (NW), hypothalamic BOLD activity during successful task performance was negatively linked to sleep efficiency (*r* = −0.68 and *p* < 0.001). This was not the case for SD (*r* = 0.02 and *p* = 0.92) and NP (*r* = −0.37 and *p* = 0.05, not significant after correction for multiple comparisons, medium effect size) conditions (Fig. 3).

Our data corroborate previous findings demonstrating an association between circadian wake promoting strength and hypothalamic BOLD activity during successful task performance in the evening hours under sleep pressure levels which are reached after a classical waking day^23^. Unexpectedly, under conditions of unusual low sleep pressure, as achieved by multiple napping, the association did not reach significance. This points to a non-monotonic dose-relationship between circadian wake-promotion and hypothalamic activity during successful task performance. In fact, we have evidence that hypothalamic activity is only significantly associated with circadian wake promoting strength when built-up homeostatic sleep pressure matches the amount typically observed under normal waking conditions (i.e., under NW). Thus, our data indicate that the link between circadian wake-promotion and hypothalamic activity is fine tuned to sleep pressure levels which usually occur under conditions of a normal day-night cycle.

Our data support the suggestion that the hypothalamus integrates circadian and homeostatic influences, affecting cognitive performance. The observed region is located at the lateral border of the postero-lateral hypothalamus (adjacent to the medial and anterior part of the substantia nigra). A very similar region has been found structurally compromised in narcoleptic patients who suffer from orexin deficiency^32^. Orexin-containing nuclei in the lateral hypothalamus represent a conceivable interface of circadian and sleep homeostatic information^33^ and are crucially involved in stabilizing behavioral states including wakefulness^34^.

### Evening hypothalamic activity is associated with behavioral vulnerability to sleep loss

So far, our results suggest a weakened or even no hypothalamic contribution to circadian wake-promotion when working memory performance is tested under disproportionally high sleep pressure levels. Importantly however, a median split analysis of our data revealed that higher hypothalamic BOLD activity at the end of a normal waking day was linked to a better ability to perform under challenging sleep loss conditions (Fig. 2c, NW vs. SD *t*_(29)_ = −2.37, *p* = 0.025). Those participants who were able to keep stable or to increase performance levels from the well-rested (NW) to the sleep-deprived (SD) state presented higher hypothalamic BOLD activity under NW. This result suggests that the degree of hypothalamic contribution to circadian wake-promotion under normal sleep pressure levels predicts sleep-loss-related performance. Note that there were no significant differences in hypothalamic BOLD activity (t_(28)_ = −0.23; *p* = 0.82), when groups were based on a median split according to the change in performance between NP and NW. Thus, in line with evidence from animal research^35^, our results indicate that the observed hypothalamic activation may play a role in behavioral performance, such that those individuals with a higher activity are less susceptible to the influence of high sleep pressure during daytime.

### Limitations

Based on the used echoplanar whole brain sequence, our data do not allow a distinct allocation of activity to a specific subpart within the hypothalamus and should therefore be interpreted with caution. The hypothalamus is located next to areas known to have signal dropout issues, and is in close proximity to blood vessels and ventricles that may induce signal distortion. Still, the association between BOLD responses in the hypothalamic region and sleep efficiency during NW holds after correction for baseline activation during a 0-back condition (*p*_corr_ = 0.007; z = 3.29). Note that heart rate, acquired in a subsample of six participants 9 hours after wake up outside of the scanner, did not significantly differ between the 3-back and 0-back condition (t_(5)_ = 0.35, *p* = 0.74; mean ± standard deviation 3-back: 66.4 ± 6.65 beats per minute; 0- back 65.5 ± 10.21 beats per minute). Furthermore, activity in the reported region follows the expected shape of the hemodynamic response function. Additionally, the reported BOLD signal is proportional to the extrinsic factor of nap sleep efficiency. Nevertheless, care should be taken when reporting BOLD responses in these areas, where a precise investigation of the BOLD time course requires the use of comprehensive models of hemodynamic response and better resolution at acquisition. The addition of a hypothalamus-specific MR sequence with higher spatial resolution in future studies would help to support our findings. Furthermore, cardiac and respiratory signals, potentially tied to arousal and the processes of sleep-wake regulation, should be recorded in future studies to partial out the impact of distortions and bias from these signals (e.g., ref. 36).

## Conclusion

We have evidence that the hypothalamus integrates circadian and sleep homeostatic control of human cognitive performance. During sleep pressure levels which are usually reached under normally entrained day-night conditions, hypothalamic activity supports circadian wake-promotion during task performance. Its supportive role for daytime performance weakens considerably when sleep pressure is below (<2 hours to last sleep opportunity) or beyond (>37 hours to last sleep opportunity) normal levels. Furthermore, hypothalamic activation during the WMZ after a normal waking day was associated with behavioural vulnerability to sleep loss. In conclusion, our data support the existence of a continuous exchange of information between homeostatic sleep pressure states and circadian clock outputs on the cerebral modulation of higher order cognitive functions. Both the dose-response and the phase relationship between the circadian timing system and sleep homeostasis play a role in the modulation of neurobehavioral performance^37^. In our 24/7 society we often experience “long days” and irregular sleep-wake regimes with considerable consequences for our cognitive performance. Our data suggest that there is a window between too little and too much sleep for a hypothalamic contribution to successful working memory performance during maximal circadian wake promotion.

## Materials and Methods

### Participants

Volunteers were recruited via advertisements on the internet. Questionnaires screened for young (20–35 years) non-smokers with good subjective sleep quality (Pittsburgh Sleep Quality Index ≤ 5)^38^, a habitual sleep duration of 8 ± 1 h, and no symptoms of clinical depression (Beck Depression Inventory [BDI-II] < 9)^39^. Exclusion criteria comprised transmeridian flights over more than 3 time zones during three months before participation, shift work, drug consumption, current medication (except contraceptives), and a history of psychiatric or sleep disorders. Overall, 31 participants (14 male, 17 female) took part in the laboratory study (see Table S1 for demographic description). Participants were genotyped regarding polymorphisms in PERIOD3 (rs57875989; 15 PER3^5/5^, 16 PER3^4/4^) and adenosine deaminase (rs73598374; 12 G/A-, and 19 G/G-allele carriers; frequency in this sample *n*.*s*., χ^2^ = 0.21), both having previously been associated with genetic vulnerability to SD (refs 40, 41 and see also refs 42–46). Methods of genotyping are described in ref. 46 for PER3 and ref. 43 for adenosine deaminase. Based on research questions explained in refs 44–46, PER3^4/5^ carriers were excluded from study participation. All participants were medically screened by the physician in charge, underwent a toxicological check (Drug-Screen-Multi 6, Nal von minden, Germany), and spent a habituation night in the laboratory to exclude sleep disorders. Participants were asked to withhold alcohol, caffeine, and tea/theophylline consumption the week prior entrance to the in-lab part of the study. Female volunteers participated, if not taking oral contraceptives, during the luteal phase of their menstrual cycle.

### Procedure

The study was approved by the local ethics committee (Ethikkommission Beider Basel) and performed according to the Declaration of Helsinki. All study volunteers gave written informed consent before participation. We carried out a randomized controlled within-subjects design with two 40-hour conditions. In the SD condition, participants were asked to stay awake for the entire 40-hour episode. Wakefulness was verified by continuous EEG recordings. In the NP, we scheduled 10 short sleep-wake cycles, each consisting of 160 min of wakefulness and 80 min of a napping opportunity. By scheduling regular naps, we induced low sleep pressure levels throughout the course of the NP. The combination of a high and low sleep pressure condition has been successfully applied in earlier studies (e.g., refs 47 and 48) to investigate the impact of differential sleep pressure levels at different circadian phases. Both conditions were separated by a minimum of seven days and preceded by one week of a fixed sleep-wake cycle (8 h sleep per night, no napping allowed) to control for sleep pressure levels and circadian misalignment. Compliance to the regimen was verified by wrist-actimetry. Participants’ scheduled wake- and sleep times, which applied also to the laboratory part, were adapted individually to their usual preferences. Baseline and recovery-nights, each of 8 hours, preceded and followed the experimental conditions. Over the course of both protocols, light was dimmed to <8 lux during wakefulness (and 0 lux during napping), meal intake was regularly scheduled (snacks every 4 hours), and body posture was controlled (semi-recumbent during wakefulness, recumbent during naps and functional magnetic resonance imaging (fMRI) sessions), to control for potential masking effects. Participants were allowed to get up only for regularly scheduled bathroom visits (equally distributed through both protocols) and did not have any time of day indication. They were allowed to read, watch documentaries or play dice-games. Social activities were restricted to communication with the study helpers. At regular intervals we assessed subjective sleepiness with the Karolinska Sleepiness Scale^49^, WM performance as well as underlying cerebral correlates, along with collecting saliva for melatonin assay. Here, we focus on BOLD activity patterns of WM performance in the first evening of the NP condition (13 hours after scheduled wake-up from the baseline-night, on average at 8 p.m.), and in the first and second evening of the SD condition (13 and 37 hours after scheduled wake-up, again at around 8 p.m.). These time windows reflect intervals of high circadian wake-promotion, as illustrated in Figure 1a by the course of sleep efficiency. By timing the fMRI to the late evening hours shortly before habitual bedtime, we targeted the WMZ^3^, characterized by maximal strength of circadian wake-promotion^4^. The fMRI sessions of 30 participants (of N = 31) in the late evening took place before the individuals’ evening onset of melatonin secretion, defined here as 50% of the maximum as previously published^43^. The evening onset of melatonin secretion has been associated with the opening of the gate for sleep^25^ and might be considered as marker of the end of the WMZ. Thus, we were able to investigate cerebral correlates of WM during high circadian wake-promotion systematically under normal, high and low sleep pressure.

### N-back paradigm

WM performance was assessed by a n-back task of about 20 min every four hours, starting one hour after waking-up from the baseline night. Every other time, participants performed the task in a MRI scanner to assess cerebral activity. The n-back task consisted of the visual presentation of a series of letters. Participants were asked to indicate by a button-press whether the letter presented is the same as n trials before. We implemented 9 blocks of a 3-back (high WM load) and 5 blocks of a 0-back condition (no WM load). In the latter, participants were instructed to press a button as soon as a specific letter appeared. Per block, a series of 30 letters comprising ten targets was presented, each for 1.5 sec with an inter-stimulus interval of 500 ms. Blocks were separated by a break of a randomly generated interval of 10–20 s, during which a fixation cross was displayed on the screen. To prevent baseline differences, participants were trained in 3-back performance in the evening before the study until they reached 70% of correct responses. Nonetheless, one participant performed three interquartile ranges below the 25^th^ percentile during the entire course of the nap condition. This performance was considered as extreme value^50^ and the data (including cerebral correlates) were excluded from further analyses.

For analyses, we focused on hit targets in the 3-back condition (true positive answers). Note that hit targets negatively correlated with false alarms (false positive answers, *p* < 0.05) and can thus indeed be considered as a measure of successful WM performance. Performance during the three sleep pressure conditions was analysed with a general linear model for repeated measures by the SPSS software (IBM SPSS Statistics for Windows, Version 22.0. Armonk, NY, USA). Degrees of freedom of *p*-values were corrected according to Huynh-Feldt, and *post*-*hoc* comparisons were assessed by repeated linear contrasts.

### Sleep EEG

Polysomnographic signals were recorded with sintered MRI compatible Ag/AgCl ring electrodes with a 15 kOhm resistor (EasyCap GmbH, Germany) and V-Amp digital sleep recorders (Brain Products GmbH, Germany). All signals were sampled at 500 Hz and filtered online by a notch filter (50 Hz). Frequencies <0.1 Hz and >20 Hz were filtered out offline (bandpass filter, butterworth type, third order, slope −60 dB/decade) for a better visual scoring. Manual sleep stage scoring of the nap sleep episodes was done according to standard criteria^51^ by experienced staff. Sleep efficiency was calculated as the percentage of the sum of the duration of all sleep stages (stage 1, 2, 3, 4, and REM) per nap duration (80 min). Here, we focus on sleep efficiency assessed in the late evening nap (on average from 9 p.m. to 10:20 p.m.) of the first day during NP.

Calculation of spectral power in the EEG delta (0.7–4 Hz) range during visually scored NREM sleep in baseline and recovery nights was performed by the ASEEGA package for the analyses of electrophysiological sleep recordings (ASEEGA, Version 3.35.11, Physip, France^52^, please see supplemental material for further information on spectral power in the EEG delta (0.7–4 Hz) range during automatically scored NREM sleep). This algorithm was used to benefit from automatic artefact rejection, but also in order to verify that a potential bias in visually scored sleep stages did not affect the results (see supplemental results). NREM sleep was considered as sum of sleep stages 2, 3 and 4. EEG power of the central bipolar derivation (Cz-Pz) as well as on the monopolar central frontal derivation (Fz) was calculated using a fast Fourier transform with a Hanning window for consecutive 30-sec artefact-free epochs. For analyses, an ANOVA for repeated measures was computed using SPSS (IBM SPSS Statistics for Windows, Version 22.0. Armonk, NY, USA), with the factors condition (NP vs. SD) and night (baseline vs. recovery). Overall, data of ten nights were lost due to technical problems (four in the NP baseline, three in the SD baseline, two in the NP recovery, and one in the SD recovery night).

### Melatonin assays

Salivary melatonin levels were analysed by a direct double-antibody radioimmunoassay (validated by gas chromatography-mass spectroscopy with analytical least detectable dose of 0.65 pm/ml; Bühlmann Laboratories, Schönenbuch, Switzerland). A bimodal skewed baseline cosine function^53^ was fitted to raw values as described in ref. 54 using MATLAB (MathWorks, Natick, MA). The evening onset of melatonin secretion was defined as the time when the melatonin level crossed 50% of the maximum at the rising limb of the curve^55^. The latter served as marker of circadian phase position^56^.

### Acquisition and analyses of fMRI data

Functional MRI images were assessed with a 3 Tesla MR Scanner (MAGNETOM Verio, Siemens Healthcare) using a standard twelve-channel head coil. Multislice T2*-weighted fMR images were acquired with a gradient echo-planar sequence applying axial slice orientation (32 slices; voxel size: 3 × 3 × 3 mm³ with 17% interslice gap; matrix size 76 × 76 × 32; repetition time = 2200 ms; echo time = 32 ms; flip angle = 82°). For anatomical reference, structural T1-weighted images were obtained with a magnetization-prepared rapid gradient echo (MPRAGE) sequence (repetition time = 2000 ms, echo time = 3.37 ms, flip angle = 8°, field of view = 25.6 cm, matrix size = 25.6 × 25.6 × 17.6 cm^3^, voxel size = 1 × 1 × 1 mm^3^). 176 contiguous axial slices covering the entire brain were assessed in sagittal direction. Due to technical problems, 3 out of 93 datasets were lost (all assessed during SD at the second evening). For analyses, we used SPM8 (http://www.fil.ion.ucl.ac.uk) implemented in MATLAB 12. Images were realigned with iterative rigid body transformations. Following normalization to the Montreal Neurological Institute (MNI) EPI template (third-degree spline interpolation; voxel size 2 × 2 × 2 mm^3^), scans were spatially smoothed (full width at half maximum 8 mm). For each subject, brain responses were then modelled at each voxel using a GLM. For each condition, the model included five regressors representing events associated with behavioural task performance (true positive, true negative, false positive, false negative responses, and events where no response was recorded). For each event type, the expected change in the BOLD signal was modelled by a canonical hemodynamic response function. Six regressors derived from realignment and a constant vector were included in the model as well and considered as variables of no interest. Family wise error (FWE) correction was applied for activations across a mask, covering brain regions typically implicated in verbal n-back performance^24^. The mask, created with MRIcron (http://www.mccauslandcenter.sc.edu/mricro/mricron/), included all coordinates listed in ref. 24 for verbal stimuli, with a radius of 10 mm.

We assessed the link between task-related BOLD activity ﻿and nap sleep efficiency (considered as a marker for circadian wake-promoting strength)^4^ by means of analyses of covariance. Sleep efficiency values were integrated as covariate of interest at the group level in the model investigating brain activity under differential sleep pressure levels, i.e., NW (after 13 hours of continuous wakefulness, normal sleep pressure level), NP (2 hours of continuous wakefulness; low sleep pressure level) and SD (37 hours of continuous wakefulness, high sleep pressure level). Inferences were based on a level of *p*_FWE_ < 0.05 with regard to a set of a-priori defined regions of interest (bilateral hypothalamic (x = 6/−6, y = −6, z = −12) and brainstem region (x = 4/−4, y = −32, z = −18) according to ref. 23) integrated into a mask by MRIcron (radius: 8 mm^23^)). We controlled for the accumulation of type 1 errors arising from three analyses (NW, NP, and SD) according to Bonferroni (*p*-level was accordingly considered as significant at *p* < 0.05/3 = 0.017).

In a final step, we were interested in whether regions associated at the BOLD activity level with circadian wake-promotion are linked to WM performance under different sleep pressure levels. Therefore, we calculated performance differences from NW (after 13 hours of wakefulness) to SD (after 37 hours of wakefulness; same circadian phase) and grouped participants based on a median split, resulting in a group with decreasing and a group with stable or even increasing performance after SD. Group differences in BOLD activity were statistically tested by a two sample t-test, using SPSS (IBM SPSS Statistics for Windows, Version 22.0. Armonk, NY, USA).

### Data availability

The datasets analysed during the current study are available from the corresponding author on reasonable request.

## Electronic supplementary material

Supplementary Information
