# Supplementary material for: Cognitive brain responses during circadian wake-promotion: evidence for sleep-pressure-dependent hypothalamic activations
Source: Sci Rep. 2017 Jul 17;7:5620. doi: 10.1038/s41598-017-05695-1 (PMC5514145; doi:10.1038/s41598-017-05695-1)
Supplement: Supplementary file 1 — Supplementary Information [file 41598_2017_5695_MOESM1_ESM.pdf]

TITLE: Cognitive brain responses during circadian wake-promotion: evidence for sleep-pressure-dependent hypothalamic activations

AUTHORS: Carolin F. Reichert[1,2], Micheline Maire[1,2], Virginie Gabel[1,2], Antoine U. Viola[1,2], Thomas Götz[3], Klaus Scheffler[4], Markus Klarhöfer[5], Christian Berthomier[6], Werner Strobel[7], Christophe Phillips[8], Eric Salmon[8], Christian Cajochen[1,2,#,*], Christina Schmidt[1,2,8,#]

Supplemental Tables.

Variable	*M*	*SD*
**Age (years)**	24.68	3.31
**PSQI**	3.13	1.18
**BDI**	1.87	2.23
**BMI**	22.21	2.49
**Wake-up time (h:min)**	07:10	00:51
**ESS**	4.21	2.46
**HO**	55.29	9.74
**MCTQ MSFsc**	4:20	1:10
**MCTQ MSFsac**	7:30	2:30

Table S1. Demographic information. PSQI = Pittsburgh Sleep Quality Index[1]; BDI=Becks Depression Inventory[2]; BMI = Body Mass Index; ESS = Epworth Sleepiness Scale[3]; HO=Horne Ostberg Morningness-eveningness Questionnaire[4]; MCTQ =Munich Chronotype Questionnaire[5]; MSFsc = Mid sleep free days corrected; MSFsac = Mid sleep free days and age corrected. N=31. SD = Standard Deviation.

**Brain area**	$\boldsymbol{T}_{1,28}$ **(peak)**	$\boldsymbol{p}_{\mathbf{FWE}}$	**X**	**Y**	**Z**
Middle frontal gyrus	7.664	<0.0001	28	4	56
Inferior frontal gyrus	6.831	0.0005	44	38	28
	8.154	<0.0001	-42	28	26
Posterior-medial frontal gyrus	10.088	<0.0001	-2	6	54
Precentral gyrus	11.348	<0.0001	-30	-4	58
Interparietal sulcus	7.717	<0.0001	34	-54	48
	8.817	<0.0001	-30	-54	48
Inferior parietal lobe	9.306	<0.0001	36	-44	40
	10.556	<0.0001	-40	-42	44
Middle occipital gyrus	8.886	<0.0001	-24	-60	40
Thalamus	5.988	0.0030	-16	-10	6
	5.314	0.0125	-12	-18	6
Cerebellum	6.357	0.0013	22	-62	-42
	6.030	0.0027	24	-68	-44
	8.172	<0.0001	-38	-58	-30

Table S2. Brain regions showing a main effect of task (BOLD signal associated with hit targets during 3-back task. The statistical t-value (one-sample t-test) of the cluster's peak voxel (T peak) is listed as well as its associated p-value (family-wise error [FWE] corrected).Coordinates (x, y, z) are expressed in mm in the Montreal Neurological Institute (MNI) space.

Brain area	$T_{1,26}$ (peak)	*p* (peak. uncorr)	X	Y	Z
**NP (positive association with sleep efficiency)**					
superior frontal gyrus	4.263	0.0001	-22	64	8
	3.767	0.0004	-16	64	16
	3.616	0.0006	-16	58	8
	3.526	0.0007	20	28	52
middle frontal gyrus	4.140	0.0001	22	50	28
precentral gyrus	3.440	0.0009	-36	-18	58
medial temporal pole	3.969	0.0002	48	10	-32
superior temporal gyrus	3.929	0.0003	-62	-16	10
middle temporal gyrus	3.837	0.0003	-50	2	-30
	3.730	0.0004	-56	-4	-26
	3.740	<0.0001	-52	0	-26
middle temporal gyrus	3.448	0.0009	-58	-60	16
inferior temporal gyrus	3.587	0.0006	-48	-12	-28
Cerebellum	4.054	0.0002	-32	-86	-30
Cerebellum	3.701	0.0005	26	-48	-32
**NP (negative association with sleep efficiency)**					
n.s.					
**NW (positive association with sleep efficiency)**					
n.s.					
**NW (negative association with sleep efficiency)**					
inferior frontal gyrus	3.676	0.0005	-54	16	28
anterior cingulate cortex	3.782	0.0004	4	34	20
caudate nucleus	3.870	0.0003	16	18	4
	3.663	0.0005	10	12	4
caudate nucleus	3.441	0.0009	-12	12	2
thalamus	3.808	0.0003	4	-6	8
hypothalamus	4.523	0.0000	4	-14	-12
calcarine gyrus	3.458	0.0009	22	-76	4
middle occipital gyrus	3.920	0.0002	40	-88	0
**SD (positive association)**					
inferior temporal gyrus	3.824	0.0004	-46	-4	-30
cerebellum	3.923	0.0003	10	-62	-46
**SD (negative association with sleep efficiency)**					
n.s.					

Table S3. Brain areas associated with evening sleep efficiency according to the sleep pressure condition. The statistical t-value of the cluster's peak voxel (T peak) is listed as well

as its associated p-value of the covariance analysis which was performed to detect regions whose BOLD signal is significantly associated with evening nap sleep efficiency during hit targets of the 3-back. Coordinates (x, y, z) are expressed in mm in the Montreal Neurological Institute (MNI) space.

Supplemental methods: Sleep EEG.

As mentioned in the main text, artefact rejection and calculation of spectral power EEG delta (0.7-4 Hz) range in artefact-free epochs during visually scored NREM sleep in baseline and recovery nights was performed by the ASEEGA package for the analyses of electrophysiological sleep recordings (ASEEGA, Version 3.35.11, Physip, France). Note that, even though initially blinded, an experienced scorer would still be able to detect whether he/she was scoring a baseline or a recovery night from sleep loss and this might induce a scoring bias. The same analysis was therefore also performed based on automatic scores, generated by the ASEEGA software. Note that for the identification of NREM sleep (sleep stage of interest for EEG delta activity), on a total of 107,453 scored 30s-epochs, an agreement of 87.0% with a Cohen's kappa coefficient of 0.71 was observed between the visual and automatic scores. In agreement with the analysis using visual scores and as depicted in Table S4, the increase of sleep pressure during SD compared to NP was manifested in a significant increase in EEG delta activity during non-rapid eye movement (NREM) sleep preceding and following each condition (interaction night (preceding baseline vs following recovery) x condition (SD vs NP):

		**Visual scoring**		**Automatic scoring**	
		**Fz**	**CzPz**	**Fz**	**CzPz**
	F	21.9	12.10	11.963	25.04
	p	< 0.0001	< 0.05	< 0.005	<0.0001
**Baseline**	NP	208.7±124	59.4±27	229.68±135	64.0±29
	SD	199.1±96	64.1±21	228.2±110	65.1±32
**Recovery**	NP	179.6 ± 72	51.3 ± 22	235.7 ± 151	61.5 ± 28
	SD	314.5 ± 176	83.3 ± 32	385.3± 139	96.3± 38

Table S4. Comparison of results assessed by visual and automatic scoring. F and *p*-values refer to repeated measures ANOVAs with the conditions night (baseline vs. recovery) and condition (multiple napping [NP] vs. sleep deprivation [SD]), performed separately for each scoring modality and electrode. Mean ± standard deviations in μV^2 are depicted by night and condition for scoring modality and derivation separately.

Finally, an ANOVA, including the factors “condition [NP, SD]”, “night” [baseline, recovery] and “analysis type” [visual, automatic] revealed that the interaction between these factors was not significant (interaction condition*night*analysis type: $F_{(1,23)} = 1.2$; $p = 0.28$).

References

1 Buysse, D. J., Reynolds, C. F., 3rd, Monk, T. H., Berman, S. R. & Kupfer, D. J. The Pittsburgh Sleep Quality Index: a new instrument for psychiatric practice and research. *Psychiatry Res* **28**, 193-213 (1989).

2 Beck, A. T., Steer, R. A. & Brown, G. K. *BDI-II, Beck depression inventory: manual.* 2nd edn, (Harcourt Brace, 1996).

3 Johns, M. W. Reliability and factor analysis of the Epworth Sleepiness Scale. *Sleep* **15**, 376-381 (1992).

4 Horne, J. A. & Östberg, O. A self-assessment questionnaire to determine morningness-eveningness in human circadian rhythms. *Int J Chronobiol* **4**, 97-110 (1976).

5 Roenneberg, T., Wirz-Justice, A. & Merrow, M. Life between clocks: daily temporal patterns of human chronotypes. *J Biol Rhythms* **18**, 80-90 (2003).
